# Single puncture percutaneous nephrolithomy for management of complex renal stones

**DOI:** 10.1186/1756-0500-2-62

**Published:** 2009-04-20

**Authors:** Mahmoud M Shalaby, Medhat A Abdalla, Hassan A Aboul-Ella, Abdel-Monem A El-haggagy, Alaa A Abd-Elsayed

**Affiliations:** 1 Urology Department, Assiut University Hospital, Assiut, Egypt; 2 Public Health and Community medicine Department, Assiut University, Assiut, Egypt

## Abstract

**Background:**

The purpose of this report is to assess the safety and efficacy of single lower pole access for multiple and branched renal calculi. A prospective non randomized clinical study included 26 patients with complex renal stones (9 patients had branched renal stones and the other 17 had multiple renal stones) in the period from May 2003 to May 2004. Mean patient age was 42 years ± 13.2 (range 18 to 67 years). All patients underwent percutaneous nephrolithotomy (PCNL) via a single lower calyceal puncture. Small stones were intactly extracted by a range of stone graspers while large stones (smallest diameter more than 1 cm) were disintegrated using either the pneumatic EMS Swiss lithoclast or Holmium YAG laser. Flexible nephroscope was used for stones inaccessible by the rigid instruments.

**Findings:**

Overall stone-free rate was 74.8%. Patients with residual stones were managed by one session of shock wave lithotripsy (SWL). Mean operative time was (80 minutes ± 27.4) for branched stones and (49.1 minutes ± 15.9) for multiple stones. No significant blood loss reported. Perforation of pelvicalyceal system occurred in 2 patients (11.5%) with no serious sequelae. Only 1 patient developed secondary hemorrhage which necessitated blood transfusion and selective angio-embolization.

**Conclusion:**

In our hands, the efficacy and safety of single lower calyceal puncture PCNL in management of complex renal stones are comparable to those of the general procedure stated in literature.

## Introduction

Renal stone disease is a challenging problem in urologic practice especially in our locality because of large stone burden and recurrence. The goal of stone treatment is to use a less morbid, minimally invasive and effective modality [1]. The management of stone disease has witnessed a revolution since the introduction of shock wave lithotripsy (SWL) and percutaneous nephrolithotomy (PCNL) [2,3]. The refinement of nephroscopes, the introduction of simple, commercially available nephrostomy sets and development of stone disintegration techniques have paved the way of PCNL [4]. After the introduction of SWL; PCNL became the spare wheel and was mostly indicated when SWL was likely to fail as in patients with large stone burden or stag horn calculi [4].

In a trial to cut the renal trauma to the minimum, we evaluate the efficacy and safety of PCNL through a single lower pole puncture for management of branched and multiple renal calculi.

## Patients and methods

After approval of the study by the ethical committee of the Faculty of Medicine, Assiut University, the study was carried out in a prospective non randomized fashion. A total of 26 patients (21 males and 5 females) with complex (branched or multiple) renal calculi among those admitted to Urology Department, Assiut University Hospital in the period from May 2003 and May 2004 were included. Mean patient age was 42 years ± 13.2 (range 18–67 years). Eight patients had pervious open surgery for renal stones. Characters of renal units and stones are presented in table 1.

**Table 1:** Characters of renal units and stones

**Variable**	**Branched stones** **(n = 9)**	**Multiple stones** **(n = 17)**
Pelvicalyceal system:		
Normal	2	6
Hydronephrotic	4	5
Pyelonephriitic	3	5
Malrotated	-	1
Previous renal surgery for stones	3	5
Radio-opaque stone	8	12
Stone burden in cm.	4.4 – 11	2.5 – 6.6
Mean (± SD) cm.	8.06 (± 2.19)	3.82 (± 1.32)

Stone distribution:		
Pelvic	-	5
Calyceal	-	2
Pelvicalyceal	9	10

Nine patients had branched renal stones (defined as stones occupying the renal pelvis and one major calyx at least) and the other 17 had multiple renal stones. All patients underwent single lower calyceal puncture PCNL.

Inclusion criteria were patients with branched and multiple renal calculi, with stone burden ≥ 2.5 cm where stone burden was measured as the sum of the largest linear dimensions of all stones based on KUB films. All patients had normal preoperative coagulation profiles and hemoglobin level greater than 11 g/dl before procedure. All procedures were performed by the same urologic team. Exclusion criteria were general contraindications of surgery, uncorrectable coagulation disorders, azotaemia and extreme obesity (body weight > 130 Kg.).

## Technique

The entire procedure was carried out under general anesthesia and fluoroscopic control. Intravenous prophylactic Cefoperazone sodium 1 gm. was administrated one hour preoperatively. The patient was placed in lithotomy position. A 6 Fr. ureteral catheter was introduced followed by retrograde pyelography to visualize the pelvicalyceal system.

The patient was then rotated to 30° oblique prone position.

Precise puncture through the proper calyx was controlled by fluoroscopy. Successful puncture was verified by free passage of saline or methylene blue then a guide wire was introduced through the 18 gauge puncture needle. Either Teflon coated J tip or Landerquist stiff guide wire were used. A safety guide wire was used in 15 procedures and was inserted through a double lumen catheter.

Alken's telescopic dilator system was used to dilate the tract up to 30 Fr. followed by introduction of an Amplatz sheath. Rigid nephroscopes were used (Wolf 24 Fr. with 25° lens or Storz 26 Fr. with 0° lens). Stones were disintegrated by standard pneumatic EMS Swiss lithoclast then extracted. Flexible nephroscope (Wolf Flexible Fibre nephroscope) was used for retrieving stones, not accessible by the rigid one, by stone basket or in-situ disintegrated using Holmium YAG laser fiber. Careful systematic inspection of the pelvicalyceal system was performed to exclude the presence of any residual stones or injury to the mucosa. The kidney was scanned by fluoroscopy (or ultrasound in case of radiolucent stones) to exclude residual stone fragments followed by retrograde pyelography with closure of Amplatz sheath to check the integrity of the pelvicalyceal system. At the end of the procedure, 22 Fr. Nelaton catheter was inserted through the Amplatz sheath to the renal pelvis.

We considered a renal unit to be free of stones if its postoperative KUB film showed no residual or insignificant residual stone material < 4 mm in radio-opaque stones, while in radiolucent stones the same criteria were applied to non-contrast computed tomography (CT) or abdominal ultrasonography (US).

Follow up visits were scheduled to be at one week after the procedure, one month then every 3 months for a minimum of 18 months.

In each visit, a thorough clinical examination, urinalysis, urine culture and sensitivity, renal ultrasonography and KUB film were performed. Excretory urography was carried out when indicated.

Statistical analysis was done by the commercially available software SPSS version 11.5. Correlations were tested using Spearman or Pearson correlation coefficients. P value of ≤ 0.05 was considered significant, ≤ 0.001 was considered highly significant and (R) = Correlation coefficient.

## Results

The overall stone free rate was 74.8%, 55.5% for branched stones and 94.1% for multiple stones. Residual stone burden ranged from 0.7 to 1.5 cm for branched stones and 0.6 to 0.8 cm for multiple stones and there was a statistically significant positive correlation between stone burden and presence of residual stones as with increased stone burden there was an increase in the residual stones (P < 0.01 and r = 0.402). Tables 2 and 3 present the relation between residual stones, stone location and stone burden.

**Table 2:** Residual stones in relation to stone location.

**Stone location**	**Pelvis**	**Upper calyx**	**Middle calyx**	**Lower calyx**
No.	22	12	14	21
Residual fragments	1	0	4	1
% Cleared	95.5	100	71.4	95.2

**Table 3:** Residual stones in relation to stone burden.

**Stone burden**	**2.5 to < 4.5 cm**	**4.5 to < 6.5 cm**	**6.5 to < 8.5 cm**	**8.5 to 11 cm**
No.	11	8	1	6
Residual fragments	1	1	0	4
% Cleared	90.9	87.5	100	33.3

Operative time ranged from 45 to 120 minutes for branched stones (mean 80 minutes ± 27.4.) and 25 to 75 minutes (mean 49.1 minutes ± 15.9) for multiple stones. There was a statistically significant positive correlation between stone burden and operative time in both groups as with increased stone burden there was an increase in the operative time (P < 0.01 and r = 0.862). Difference in hemoglobin levels in pre and immediate postoperative blood counts were considered as indicator of intra-operative blood loss. The estimated intra-operative drop in hemoglobin (Hb) level ranged from 0.3–2 g/dl (mean 0.52 g/dl) for branched stones and from 0.2 – 2.8 g/dl (mean 0.44 g/dl) for multiple stones. There were statistically significant positive correlations between stone burden and hemoglobin drop and between operative time and hemoglobin drop as with increased the amplitude of hemoglobin drop there was and increase in both the stone burden and operative time (P < 0.01, r = 0.458 and P < 0.01, r = 0.46 respectively).

Minor intra-operative complications occurred in 3 patients: perforation of the pelvicalyceal system occurred in 2 patients (among the group with multiple stones). Hypotension occurred in 1 patient with a branched stone and was managed conservatively without blood transfusion. There were statistically significant positive correlations between stone distribution and intra-operative complication as with increased the stone distribution there was an increase in the intra-operative complications (P = 0.05, r = 392), also between operative time and intra-operative complications as there was an increase in intra-operative complications with increases operative time (P < 0.01, r = 0.425).

Post-operative complications were reported in 6 patients in the form of: secondary hemorrhage in 1 patient with multiple stones occurred in the seventh post-operative day and was managed successfully by blood transfusion followed by selective angiography and angio-embolization of bleeding lower polar segmental artery. Post-operative fever occurred in 5 patients and was managed conservatively. Pereoperative complications are summarized in table 4.

**Table 4:** Complications of PCNL

**Group Complication**	**Branched stone group (n = 9)**	**Multiple stone group (n = 17)**	**Percent**
Intra-operative:			
Perforation	0	2	3/26
Hypotension	1	0	11.5%

Post-operative:			
Low-grade fever	2	2	6/26
High-grade fever	1	0	23.1%
Secondary hemorrhage	0	1	

Overall	4/9	5/17	9/26
	44.4%	29.4%	34.6%

Percutaneous nephrostomy tube was removed after a mean of 2.58 days (range 1–5) while ureteral catheter was removed after a mean of 3.65 days (range 2–6). Hospital stay ranged from 2–10 days (mean 3.92).

All residual stones (n = 6) were within the range of 0.7–1.5 cm and were managed by a single session of SWL, except in 1 patient ureteroscopic retrieval of a lower ureteral stone fragment was needed (a total of 7 ancillary procedures).

## Discussion

Despite the introduction of SWL for treatment of renal calculi, PCNL still plays an important role in the treatment of large stone burden, radiolucent stones and stones in patients with anatomic concerns [5]. Improvement of endourologic instruments and lithotripsy devices has yielded greater success rates and lower complications rates for percutaneous renal surgery [5].

Proper access is a prerequisite for complete clearance of renal calculi by PCNL. The ideal tract is one that provides the shortest and straightest access to all calculi [6].

Subcostal lower pole punctures were performed in the current study because it has fewer complications [7,8]. Most stones resided in the lower calyx or pelvis or both. This is in agreement with the general principle for access site selection stated by Lingeman et al., that percutaneous access to the kidney should allow maximal stone removal using a rigid nephroscope [9].

The combined use of rigid and flexible nephroscopes facilitate stones retrieval through calyces that could not be negotiated by rigid nephroscope alone and the use of the flexible Holmium YAG laser fibers through flexible nephroscope help in in-situ disintegration of calyceal stones and improved the overall success rate.

In our study the overall stone-free rate was 74.8%. This rate is higher than that reported by Maghraby et al. and Singla et al. after a single session (52% and 70.7% respectively) [10,11] and lower than that reported by Holman et al. and Jou et al. (96% and 82.8% respectively) [12,13].

A total of 7 ancillary procedures were needed after 26 sessions of PCNL (26.9%) to render all our patients stone free. This rate is much higher than that reported by Albala et al. (2% for the PCNL group) [14] and less than that reported by Golijanin et al. (30.4% SWL and 15.6% second look PCNL) [15]. Six out of the 7 ancillary procedures were SWL which in our opinion is preferred to multiple tracts because of concerns regarding renal trauma and operative time.

The difference between pre and post-operative hemoglobin level was used as an indicator of blood loss, in our study no intra-operative blood transfusion was required and the mean decrease in hemoglobin level was 0.48 g/dl which was less than that reported by Shaban et al., who reported 0.79 g/dl mean hemoglobin drop [16].

The mean operative time in our study was 51.19 ± 24.39 minutes which is shorter than that reported by Kurtulus et al, who reported mean operative time of 2.3 and 2.2 hours [17].

Overall intra-operative complications were 11.5% in the form of perforation of pelvicalyceal system and hypotension. This compares favorably with that of Lingeman et al., who stated that minor intra-operative complications can be seen in 11 to 25% of patients [9].

Post-operative complication rates ranged between 3.2% as reported by Segura [18], to 12.8% as reported by Singla et al [11]. In this study, secondary hemorrhage necessitating a hemostatic maneuver occurred in 1 patient (3.8%) and was managed by selective angio-embolization for an arterio-venous fistula. This is comparable to what was reported by Gremmo et al. (2.3%) [19] and less than that reported by Jones et al (5.8%) [20].

In this study, there were no complications involving the chest cavity. Such complications are reported by almost every study in literature investigating the safety of upper calyceal approach. Singla et al. reported occurrence of hydrothorax in 7 patients and haemothorax in 1[11] while Shaban et al. had one patient who suffered from hydrothorax and another one who developed renopleural fistula [16]. Aron et al. who compared upper to lower calyceal approach in a prospective non randomized fashion, reported 2 incidents of hydrothorax that required chest tube insertion among the group with upper calyceal access compared to non in the group with lower calyceal approach.

## Conclusion

In our opinion, the single lower calyceal puncture is safer than the upper calyceal access and less traumatic compared to multiple tracts in management of complex renal stones and we recommend it especially for ESWL equipped centers.

## Competing interests

The authors declare that they have no competing interests.

## Consent

Written informed consent was obtained from all patients for publication of this manuscript.

## Authors' contributions

MS carried out the patient diagnosis, investigation, surgical management, follow up management, drafting of the manuscript and manuscript writing. MA, HA, AE carried out the patient diagnosis, investigation, follow up management. AAA-E carried out the medical management, general coordination, drafting of the manuscript, writing the final manuscript and provided important suggestions. All authors read and approved the final manuscript.
